# Wideband acoustic absorbance in children with Down syndrome^☆^^☆☆^

**DOI:** 10.1016/j.bjorl.2017.12.006

**Published:** 2018-01-10

**Authors:** Alessandra Spada Durante, Mayara Santos, Nayara M.C. de F. Roque, Marcella S. Gameiro, Katia de Almeida, Osmar Mesquita de Sousa Neto

**Affiliations:** a Faculdade de Ciências Médicas da Santa Casa de São Paulo (FCMSCSP), Curso de Fonoaudiologia, São Paulo, SP, Brazil; b Faculdade de Ciências Médicas da Santa Casa de São Paulo (FCMSCSP), Curso de Medicina de Otorrinolaringologia, São Paulo, SP, Brazil

**Keywords:** Middle ear, Down syndrome, Acoustic impedance tests, Orelha média, Sindrome de Down, Testes de impedância acústica

## Abstract

**Introduction:**

Tympanometry is currently the most frequently used tool for assessing the status of the middle ear, commonly assessed using a single 226 Hz tone. However, the use of the Acoustic Immittance Measures with a wideband stimulus is a promising high-resolution evaluation, especially in individuals known to have middle ear alterations, such as Down syndrome patients.

**Objective:**

The aim of this study was to analyze the acoustic absorbance measurements in children with Down syndrome.

**Methods:**

Cross-sectional study, approved by the institution's ethics committee. Data were collected from 30 children, with a mean age of 8.4 years, 15 with Down syndrome (DS-study group) and 15 children with typical development and no hearing complaints (control group). Energy absorbance was measured at frequencies of 226–8000 Hz at ambient pressure and at peak pressure as a function of frequency using TITAN equipment. Statistical analysis was performed using the established level of statistical significance of 5%.

**Results:**

With the 226 Hz probe tone, 30 ears of the control group and 22 of the study group exhibited Type A tympanograms, whereas Type B was observed in eight children in the study group. The mean acoustic absorbance ratio of the study group was lower than that of the control group at frequencies centered at 2520 Hz (*p* = 0.008) for those with normal tympanometry results, and 226–4000 Hz (*p* < 0.03) for those with a Type B tympanometry curve.

**Conclusion:**

The low energy absorption in the presence of normal tympanograms in children with Down syndrome may suggest middle ear abnormalities.

## Resumo

**Introdução:**

A timpanometria é a ferramenta mais usada para avaliar o status da orelha média, comumente avaliada por meio de uma única frequência com o tom de 226 Hz. No entanto, o uso da medida de imitância acústica com estímulo de banda larga é uma avaliação de alta resolução promissora, especialmente em pacientes conhecidos por frequentemente apresentar alterações da orelha média, como a na síndrome de Down.

**Objetivo:**

Analisar as medidas de absorvância acústica em crianças com síndrome de Down.

**Método:**

Estudo transversal, aprovado pelo comitê de ética da instituição. Foram coletados dados de 30 crianças, com idade média de 8,4 anos, sendo 15 com síndrome de Down (SD-grupo de estudo) e 15 crianças com desenvolvimento típico e sem queixas auditivas (grupo controle). A absorvância de energia foi medida nas frequências de 226-8.000 Hz à pressão ambiente e no pico de pressão em função da frequência, usou-se o equipamento Titan. A análise estatística foi feita com o nível de significância estatística adotado de 5%.

**Resultados:**

Com o tom de sonda de 226 Hz foram observadas 30 orelhas do grupo controle e 22 do grupo estudo com timpanometria Tipo A e o Tipo B foi observado apenas em oito crianças do grupo estudo. A razão média de absorvância acústica do grupo estudo foi menor que a do controle nas frequências centradas em 2.520 Hz (*p* = 0,008) para aqueles com resultados timpanométricos normais e de 226-4.000 Hz (*p* < 0,03) para aqueles com curva timpanométrica Tipo B.

**Conclusão:**

A baixa absorção de energia na presença de timpanogramas normais nas crianças com síndrome de Down pode sugerir anormalidades na orelha média.

## Introduction

Down syndrome (DS) is a genetic condition, caused by an extra chromosome in pair 21. The presence of an extra chromosome occurs by free trisomy in 96% of the cases, in which there is a non-separation of the pair of chromosomes 21 during the meiosis of one of the parents, resulting in the existence of an ovum or sperm cell with 24 chromosomes. The estimated incidence of Down syndrome is 1 in 1000 live births worldwide, making it the most common of the genetic syndromes. Common manifestations of this syndrome include auditory abnormalities including low-set earlobes and external auditory meatus (EAM) stenosis, characteristics that may result in the accumulation of cerumen in the EAM when associated with smooth muscle hypotonia. Affected individuals also possess a more horizontal Eustachian tube and experience frequent upper airway infections, which frequently result in a middle ear fluid accumulation and suppuative otitis. Presbycusis has also been described in adolescents with DS, due to the early aging in DS individuals.^1^, ^2^ It has been estimated that 60–80% of children with DS have some type of hearing loss with a significant risk of otitis media (50–70%), which often makes early diagnosis^3^ of neurosensory hearing loss^4^, ^5^ difficult.

Improved understanding of medical problems along with recent and more consistent approaches in the medical and surgical care of otorhinolaryngological manifestations associated to DS have led to important improvements in the health care of these children. The current health care guidelines for children with DS, as prescribed by the American Academy of Pediatrics Committee on Genetics,^3^ recommend audiological tests at birth, and monitoring every 6 months up to the age of 3 years, with annual tests after 3 years of age, including tympanometry measurements to better identify middle ear alterations.

However, cognitive issues are challenging and have an impact on the results of audiological assessments in both behavioral measures and in the performance of objective tests, such as the recording of otoacoustic emissions.^6^, ^7^ Even conventional tympanometry can be difficult to perform, due to the frequent narrowing of the external auditory meatus.^1^ Recently, wideband tympanometry has emerged as a procedure capable of detecting mild middle ear alterations and consequent otitis media in children and neonates, with greater sensitivity and specificity than conventional measures of tympanometry with a 226 Hz probe tone.^8^, ^9^, ^10^, ^11^

Only three studies in the literature investigated wideband tympanometry in children with DS; two studies used acoustic reflectance measurements^12^, ^13^ and one recent study used acoustic absorbance measurements. It highlighted specific patterns in the curves of children with DS, which makes this measure promising in the diagnosis of conductive problems by providing frequency-specific information.^14^

The aim of this study was to analyze acoustic absorbance with wideband stimuli at ambient pressure and peak pressure in children with Down syndrome, compared to children with normal development, employing Jerger's classification of the tympanometry curves, with a 226 Hz probe tone in the right and left ears, at 17 frequencies, between 226 and 8000 Hz.

## Sample and methods

This research was approved by the institution's Ethics Committee (345/15). All subjects participated after their parents/legal guardians were informed and signed the Free and Informed Consent Form. This is a prospective, cross-sectional and analytical study.

### Sample

A total of 30 children participated in the study, with 15 in the control group, of which 8 were boys and 7 were girls, with ages between 6 and 10 years and mean age of 8.4 years and 15 in the study group, of which 8 were boys and 7 girls, with ages between 5 and 14 years and mean age of 8.4 years.

The inclusion criteria of the study group were children with a diagnosis of Down syndrome treated at the ISCMSP Down syndrome outpatient clinic, and for the control group, children with normal development, without auditory complaints and without auditory risk factors who were enrolled in a school in the downtown region of the city of São Paulo were matched by gender and age with the study group. Children who did not complete the research protocol were excluded from the study.

### Equipment

The Titan equipment, manufactured by Interacoustics and the IMP440 Clinical module were used, with the optional 3D Wideband tests and the WideBand Tympanometry (WBT) research modules. This module automatically extracts the values of the obtained measurements and transforms them into data in spreadsheets. Thus, for each subject, it was possible to analyze the absorbance values at the peak and the ambient pressure. Therefore, the following frequencies were selected: 226 Hz; 257 Hz; 324 Hz; 408 Hz; 500 Hz; 630 Hz; 794 Hz; 1000 Hz; 1260 Hz; 1587 Hz; 2000 Hz; 2520 Hz; 3175 Hz; 4000 Hz; 5040 Hz; 6350 Hz; 8000 Hz; totaling 17 frequencies per ear tested at ambient pressure; and 17 at peak pressure. The choice of frequencies was made according to the literature.^10^, ^15^, ^16^

### Procedures

This study investigated acoustic absorbance measurements in children with Down syndrome and children with normal development, divided according to the tympanometry curve at 226 Hz. Each group had 15 children matched by gender and with the same mean age. The examination was carried out in a private room, where the child was seated comfortably in an armchair, when necessary, together with the caregiver, to feel more relaxed and secure. The entire examination was explained to parents/caregivers, but was always directed to the child. Due to the short attention span of DS children, there were some difficulties during the examination that increased the session time by up to 30 min. Frequent reorientation was necessary, followed by strategies to relax the child, with educational toys or videos that kept the children calm and quiet, making the examination feasible.

After visual inspection of the external acoustic meatus, the olive (sealing device) was selected that best suited the size of the child's ear. The wideband tympanometry measurement took on average 10 s to be performed in each ear. From this examination, data were extracted on the tympanometry at 226 Hz and the acoustic absorbance. Children were evaluated bilaterally.

The extraction of data on acoustic absorbance was obtained using the “140331 WBT absorbances and averaged tymp” worksheet. During the extraction, an Excel spreadsheet is generated and, it was possible to visualize the values of absorbance at the peak and ambient pressure by frequency in each subject.

The Wilcoxon, Mann–Whitney, Chi-Square, Fisher and McNemar tests were used for the inferential analysis of the results, with version 13.0 of the SPSS software package. The level of significance adopted for this study was 5% (*p*-value ≤ 0.05).

## Results

The groups were homogeneous regarding gender and age (*p* > 0.05). However, due to differences for the tympanometry configuration, with a 226 Hz probe tone,^17^ analyses were performed separately comparing children with Down syndrome and Type A tympanometry curve (*n* = 7) and the control group, in which all children had a Type A curve (*n* = 15). Children with Down syndrome and Type B tympanometry curve (*n* = 8) were analyzed separately.

**Figure 1:**
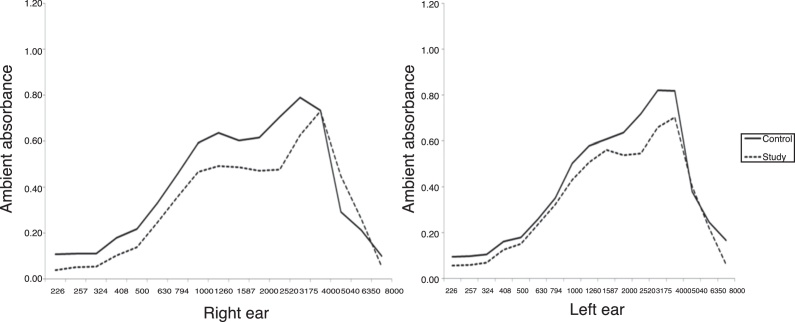
Comparison of mean acoustic absorbance values at ambient pressure per group, frequency and ear.

**Figure 2:**
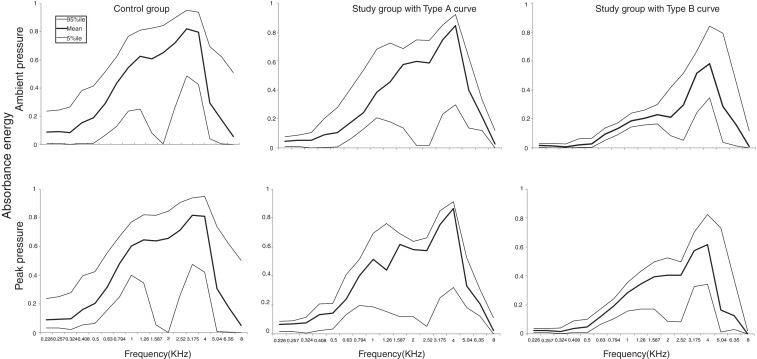
Acoustic absorbance (5% percentile, mean and 95% percentile) by frequency and group according to tympanometry configuration.

No differences were observed between the ears. The mean curves of acoustic absorbance in the group of children with Down syndrome and Type A tympanometry curve are shown in Fig. 1. Fig. 2 shows the acoustic absorbance values at ambient pressure and peak pressure by group, frequency and type of tympanometry curve. The 95th, 50th, and 5th percentile values are highlighted. The mean energy absorption ratio of the DS group was lower than that of the control group at frequencies centered at 2520 Hz (*p* = 0.008) for those with normal tympanometry results, and at 226–4000 Hz (*p* < 0.03) for those with type B tympanometry curve, both at ambient pressure and at peak pressure.

## Discussion

The importance of studies that address objective hearing assessment techniques in individuals with Down syndrome is justified by the difficulty in establishing reliable behavioral thresholds. In the literature, several researchers point out that this difficulty exists in up to 62.5% of ears.^6^, ^7^, ^12^

There is a high occurrence of middle ear disorders in DS, but there have been few studies that used wideband acoustic immittance measurements, since this technology has been used in this population very little.

The wideband tympanometry measure makes it possible to verify the middle ear status, as it can disclose the nature of an alteration through the magnitude and latency of the acoustic energy that is absorbed or reflected by the tympanic membrane as a function of frequency. Both measures are complementary and can be observed in percentage charts or on a scale of 0.0–1.0.^18^ In the case of this study, the percentage chart of acoustic absorbance was observed during the test performance, on the Titan equipment screen; however, when extracting the values into the Excel file, the values were transformed into a linear scale (from 0.0 to 1.0).

In the present study, regardless of the tympanometry findings, the data showed reduced wideband energy absorbance in children with DS. The mean energy absorption ratio for the DS group was lower than that of the control group at frequencies centered at 2520 Hz (*p* = 0.008) for those with normal tympanometry results, and at 226–4000 Hz (*p* < 0.03) for those with Type B tympanometry curve, at both ambient and peak pressure.

Through wideband reflectance at ambient pressure, Kaf^12^ (2011) observed that there was an abnormally smaller difference in the reflectance curve of the study group compared to the reflectance measures of the control group. This abnormal difference existed when comparing subjects with normal tympanometry in the control group and subjects with abnormal tympanometry in the study group. Similarly, in the present study, the absorbance curve was smaller when compared with that of the control group, in which all had tympanometry curves considered normal. The same author also observed that children with normal tympanometry on the 226 Hz probe had abnormally low reflectance ratios above 4000 Hz, which could suggest associated congenital middle ear anomalies in these children with Down syndrome. In the present study, this difference was also identified for frequencies centered at 2520 Hz when the two groups had normal tympanometric patterns.

The study by Soares (2016)^13^ characterized the response curve of the middle ear in Down syndrome through wide-frequency reflectance, where children with Down syndrome and normal hearing showed a reflectance curve similar to that of the control group. In this study it was observed that children with Down syndrome and intact middle ear had an absorbance curve similar to the curve observed in the control group, in which all the children had normal tympanometry curves.

A recent study that assessed children with DS (*n* = 40, mean age 6.4 years) and children with normal development (*n* = 48, mean age 5.1 years), also demonstrated that wideband absorbance was significantly lower at 1–4 kHz only in the ears with conductive loss compared to ears with normal hearing for both groups.^14^

Studies with wideband tympanometry measurements have shown great applicability in the identification of middle ear conduction alterations,^10^, ^19^, ^20^, ^21^, ^22^ which is desirable in the assessment of populations that are more prone to such alterations.^4^ This middle ear assessment technique has some advantages, such as covering a wide range of frequencies, not necessitating such a critical positioning of the probe in the external auditory meatus, especially at high frequencies and not requiring pressurization of the external acoustic. It requires only a short time to perform a full absorbance scan over a wide frequency range, and is a very sensitive test in the evaluation of middle ear alterations and conductive hearing loss. Absorbance measurements are performed at ambient pressure or peak pressure. In this study, we chose to perform the analysis at both ambient pressure and peak pressure. In the present study, all absorbance values for the ambient pressure and peak pressure showed the same intra-group curve characteristics, with lower absorption at low frequencies and higher absorption at high frequencies. This factor is due to mass and rigidity effects. Statistically significant differences were observed between pressurized and ambient responses only in the presence of middle ear alterations, as in the study by Keefe and Simmons (2003),^19^ in which the peak pressure absorbance was more accurate for a correct diagnosis than the ambient pressure. However, Liu et al. (2008)^23^ when assessing the absorbance energy in adults with normal hearing, found similar peak pressure values at different scanning velocities, but higher values below 2 kHz than the absorbance measured at ambient pressure.

In this study, there was no significant difference between the measurements of the left and the right ears, as reported by Shahnaz and Bork (2006),^15^ Hunter et al. (2008).^9^

As for the absorbance values, according to the literature, one would expect lower values for the low frequencies and higher for the medium and high frequencies. Values should be between 0.10 and 0.90 to be considered normal for the wideband (i.e., a normality between 10% and 90%). Values outside this area of normality can be suggestive of conductive hearing loss secondary to, for example, secretory otitis media, a prior tympanoplasty, or a Type C tympanometry curve with 226 Hz probe, as in the case described by Stanford and Brockett (2014).^24^ It is believed that low values for low frequencies can be explained by EAM sealing with the incorrect device^9^, ^25^ or the presence of fluid in the outer ear or middle ear. In the present study, the measurements were carried out under a strict protocol, so that the adequate EAM sealing was available. Thus, in the DS group with Type A tympanograms, these results are believed to reflect the peculiar anatomical condition of patients with DS.

When comparing the absorbance of children with Down syndrome and type A tympanometry curve with the absorbance of the children in the control group, the results reveal a reduced energy absorption curve, with a statistically significant difference in frequencies centered at 2520 Hz, both at ambient pressure and peak pressure. Although the sample is small, the statistical analysis results showed the typical behavior in the DS population.

Future studies could confirm the findings of the present study and broaden the assessed age groups, and contribute to a better understanding of the acoustic absorbance measures in Down syndrome as a function of aging.

## Conclusion

Low energy absorption in the presence of normal tympanograms in children with Down syndrome may suggest middle ear abnormalities.

## Conflicts of interest

The authors declare no conflicts of interest.
